# Two Cases in Practice

**Published:** 1884-04

**Authors:** J. L. Williams

**Affiliations:** New Haven, Conn.


### TWO CASES IN PRACTICE.

BY J. L. WILLIAMS, NEW HAVEN, CONN.

Miss K. called for an examination and appointment. While examining the right upper first molar I discovered a fistulous opening in the roof of the mouth, at a point about opposite the end of the palatine root of the tooth. On calling her attention to it, she said that there had been a swelling at that point about a year previous, and it had left the little opening which I had discovered. My suspicions were at once directed towards a pulpless lateral on the same side of the jaw. But a careful examination of the tooth, and also the failure of the exploring probe to find an opening in that direction, led to further consideration. All of the third molars were in position, except on this side of the upper jaw. She was certain that no tooth had been extracted in this region. A more close examination of the crown of what I had at first regarded as the right upper second molar, and a comparison of this crown with that of the third molar on the opposite side, convinced me that it was the third molar for this side. The missing tooth, then, was the second molar. Evidently here was a case where it would be judicious to "make haste slowly." I inserted the point of a stout lance-blade in the fistulous opening, and made a clean cut through to the bone and down to the margin of the gum. I should have previously remarked that the first molar was free from decay, and gave evidence of having a healthy pulp. After making the incision I explored carefully with a needle point. In the vicinity of the fistulous opening I could feel what I at first supposed was the palatine root of the first molar. But as it seemed to be a little further back than the normal position of this root would be, I determined to remove the external wall of bone from the opening down to the alveolar margin, which I did with a large rose burr. I could now trace the root down between the first and third molars, these teeth being in contact with each other. I could not understand why the second molar should have failed to erupt, or how the third molar could have pressed forward over it until it was in contact with the first molar. I packed cotton firmly into the incision, and dismissed the patient, to call the following morning.

On removing the cotton next morning I could see, by aid of the mouth mirror, the discolored root. I could also determine by the use of the needle-pointed explorer, that it had no connection with either of the other teeth. I then cut around it as well as I could by the use of fissure burrs, and passing the point of a curved elevator around it I loosened the mass, and with a slender-pointed forcep brought away—the palatine root of the second molar. Further exploration failed to detect any additional portion of the tooth. The most remarkable feature of the case was that the patient should have had no recollection of the decay of the crown and loss of the two buccal roots of the tooth. The expulsion of the roots was probably hastened by the eruption of the third molar, which pressed forward and imprisoned the palatine root. The opening healed rapidly, but has left quite a marked depression.

The case illustrates the importance of taking all the factors of a problem into consideration before attempting its solution.

Case 2. Mrs. S. had been wearing a pivot tooth on the root of the upper right central for several years, and as it required frequent resetting she desired to have it replaced by a more permanent operation. On removing the crown, the root was found in a bad condition. Decay had penetrated the side of the root, leaving quite a large opening into the pericementum. An enlarged foraminal opening led to a cavity at the end of the root, from which an offensive pus was discharged. But the root was very firm, and promised to give a secure foundation for a crown if it could be brought into a healthy condition. A little cowardice prompted me to attempt the treatment through the root, but after a week's effort my ambition in that direction was satisfied, and I resorted to a method which has proved eminently successful in several cases of this character. The end of a soft, smooth broach was bent so as to form a little hook. This was passed up the enlarged pulp canal until the hook slipped over the end of the root. The broach was then seized with pliers at a point exactly opposite the external end of the root and drawn out, and the length carefully measured.

A point of orange wood was carefally shaped to fit the pulp canal, a notch cut on one side, showing the exact length of the root inside, and after dipping in a solution composed of equal parts of carbolic acid, chloral hydrate and gum camphor, it was driven into the canal until the notch appeared precisely opposite the end of the root. I know of no other method by which an enlarged pulp canal can be so perfectly filled, with a certainty that the filling material has gone exactly to the end of the root and no further.

The wood point was twisted off at a point about half the length of the root, where it had been weakened by passing a knife around it, cutting partly through. Heavy gold foil was placed over the opening in the side of the root, and the large funnel-shaped opening filled with amalgam. An external opening was made opposite the end of the root, and the diseased bone and end of the root cut away with rose burrs. A cotton tent was kept in for two days. On the third day a crown was placed on the root, and in ten days the external opening healed, and all irritation had passed away.

